# Relationship between angiography timing and angiographic visualization of extravasation in patients with acute non-variceal gastrointestinal bleeding

**DOI:** 10.1186/s12876-020-01570-y

**Published:** 2020-12-14

**Authors:** Chungjo Choi, Hyun Lim, Min-Jeong Kim, Bo Young Lee, Sung-Yeun Kim, Jae Seung Soh, Ho Suk Kang, Sung Hoon Moon, Jong Hyeok Kim

**Affiliations:** 1 Department of Internal Medicine, University of Hallym College of Medicine, Hallym University Sacred Heart Hospital, 22 Gwanpyeong-ro 170-gil, Dongan-gu, Anyang, 431-796 Republic of Korea; 2 Department of Radiology, University of Hallym College of Medicine, Hallym University Sacred Heart Hospital, Anyang, Republic of Korea

**Keywords:** Angiography, Endovascular, Gastrointestinal bleeding, Trans-arterial embolization

## Abstract

**Background:**

Angiographic embolization is now considered the first-line therapy for acute gastrointestinal (GI) bleeding refractory to endoscopic therapy. The success of angiographic embolization depends on the detection of the bleeding site. This study aimed to identify the clinical and procedural predictors for the angiographic visualization of extravasation, including angiography timing, as well as analyze the outcomes of angiographic embolization according to the angiographic visualization of extravasation.

**Methods:**

The clinical and procedural data of 138 consecutive patients (mean age, 66.5 years; 65.9% men) who underwent angiography with or without embolization for acute non-variceal GI bleeding between February 2008 and July 2018 were retrospectively analyzed.

**Results:**

Of the 138 patients, 58 (42%) had active extravasation on initial angiography and 113 (81.9%) underwent embolization. The angiographic visualization of extravasation was significantly higher in patients with diabetes (*p* = 0.036), a low platelet count (*p* = 0.048), high maximum heart rate (*p* = 0.002) and AIMS65 score (*p* = 0.026), upper GI bleeding (*p* = 0.025), and short time-to-angiography (*p* = 0.031). The angiographic embolization was successful in all angiograms, with angiographic visualization of extravasation (100%). The clinical success of patients without angiographic visualization of extravasation (83.9%) was significantly higher than that of patients with angiographic visualization of extravasation (65.5%) (*p* = 0.004). In multivariate analysis, the time-to-angiography (odds ratio 0.373 [95% CI 0.154–0.903], *p* = 0.029) was the only significant predictor associated with the angiographic visualization of extravasation. The cutoff value of time-to-angiography was 5.0 h, with a sensitivity and specificity of 79.3% and 47.5%, respectively (*p* = 0.012).

**Conclusions:**

Angiography timing is an important factor that is associated with the angiographic visualization of extravasation in patients with acute GI bleeding. Angiography should be performed early in the course of bleeding in critically ill patients.

## Background

Acute gastrointestinal (GI) bleeding is a medical emergency with a mortality rate ranging from 8 to 14% [1–3]. After initial assessment and hemodynamic resuscitation, urgent endoscopy is the treatment of choice for patients with acute GI bleeding [4–6]. However, severe bleeding despite endoscopic therapy occurs in 5–10% of patients, and the management for sustained bleeding after failure of endoscopic therapy remains a significant clinical challenge.

Surgery and angiographic embolization are the available treatment options for patients with acute GI bleeding refractory to endoscopic therapy. However, to date, no controlled trial has compared angiographic embolization to surgery as a salvage procedure for patients with acute GI bleeding refractory to endoscopic therapy. Surgery is generally an expeditious and desirable approach to achieving favourable outcomes; however, it can be associated with a high operative mortality rate of 25% [7, 8]. Compared with surgery, angiographic embolization controls acute GI bleeding in a high proportion of patients including the critically ill and those who had previously undergone surgery [9, 10]. Hence, this technique is now considered the first-line therapy for massive GI bleeding that is refractory to endoscopic therapy.

The success of angiographic embolization for acute GI bleeding depends on the detection of the bleeding artery site. Active contrast medium extravasation is the only direct and most common angiographic sign of acute GI bleeding, and can be treated with angiographic embolization [11, 12]. However, despite the established efficacy of angiographic embolization, the detection rate of angiographic visualization of extravasation is at 24%–78% of patients [13, 14]. Data on the angiographic visualization of extravasation have been reported in only a limited number of small series to date. Moreover, it is unclear which patients will show the angiographic visualization of extravasation. Furthermore, the association between angiography timing and angiographic visualization of extravasation has not been studied.

The aims of this study were to identify the different clinical and procedural predictors, including angiography timing, for the angiographic visualization of extravasation, as well as to evaluate the outcomes of angiographic embolization in the management of acute GI bleeding according to the angiographic visualization of extravasation.

## Methods

### Study population

Data from 138 consecutive patients (mean age, 66.5 years; 65.9% men) who underwent angiography with or without embolization for acute non-variceal GI bleeding at the Hallym University Sacred Heart Hospital, Anyang, South Korea, between February 2008 and July 2018, were retrospectively reviewed. In all patients, endoscopic therapy using injections (epinephrine or fibrin sealants), thermal devices (bipolar electrocoagulation probes), or clips had either failed or been abandoned due to the inability to control bleeding. This study was conducted in a tertiary hospital with 24-h endoscopy and angiography services for acute GI bleeding.

The clinical and procedural data reviewed included age, sex, comorbid illnesses (hypertension, diabetes, cirrhosis, chronic renal failure, chronic respiratory disease, ischemic heart disease, heart failure, cerebrovascular disease, and malignancy), use of antiplatelet agents and anticoagulants, initial laboratory findings, vital signs, risk stratification score of GI bleeding (clinical Rockall score, Glasgow-Blatchford score, and AMIS65), bleeding site and cause, time-to-angiography, angiographic findings, embolization, complications, technical and clinical outcomes, and date of death or last follow-up. We analyzed only the initial angiographic findings, comparing the clinical and procedural factors of patients with active extravasation with those of patients without active extravasation to identify predictors for an angiographic visualization of extravasation. This study was approved by our study’s institutional review board (2020-04-33) and was conducted in accordance with the Declaration of Helsinki.

### Endpoints

**Fig. 1:**
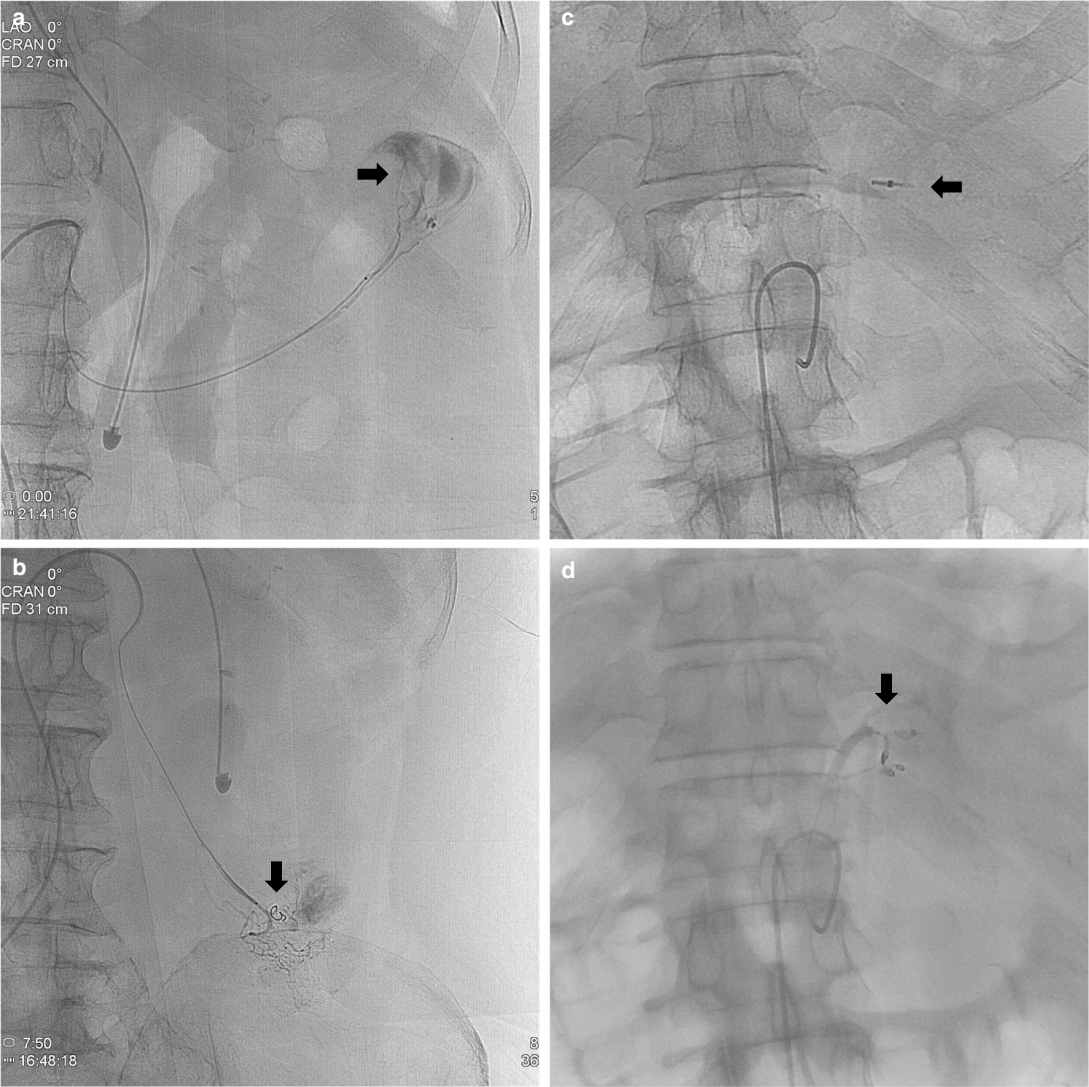
Transcatheter arterial embolization for acute gastrointestinal bleeding. **a**, **b** Contrast extravasation guided embolization. Selective angiography shows contrast medium extravasation from the jejunal branches of the superior mesenteric artery (black arrow) (**a**). After microcatheterization guided by contrast extravasation, bleeding was controlled by embolization using microcoils and gelfoam (**b**). **c**, **d** Blind embolization. Angiography before embolization shows no evidence of contrast medium extravasation (**c**). After microcatheterization guided by clip position (black arrow), the left gastric artery terminating at clip was selectively embolized using microcoils and gelfoam (**d**)

An experienced radiologist (K.M.J) retrospectively reviewed the angiograms and procedural records to search for extravasation and hemostasis in all patients. Angiography for patients with upper GI bleeding was performed via selective catheterization of the celiac artery and superior mesenteric artery (SMA), followed by super-selective catheterization of the celiac artery and SMA branches. Selective catheterization of the SMA and inferior mesenteric artery (IMA) was performed for patients with lower GI bleeding, followed by super-selective catheterization of the SMA and IMA branches. Extravasation was identified as leakage of the intravenously-administered contrast medium from the normal intravascular compartment into the bowel lumen (Fig. 1); the embolization agent was selected by the attending interventional radiologist. The radiologist also evaluated the post-embolization angiograms to determine embolization success. Indirect signs on angiography (aneurysms/pseudoaneurysms, vessel irregularity, vessel cutoff and arteriovenous/arterioportal shunting, neovascularity, and increased vascularity from dilated arterioles) were not considered as the angiographic visualization of extravasation.

### Definition

Upper GI bleeding occurs proximal to the ligament of Treitz and may involve the esophagus, stomach, and duodenum. Lower GI bleeding occurs distal to the ligament of Treitz and may involve the small bowel, colon, and rectum. Among vital signs, the lowest systolic blood pressure and maximum heart rate were defined as checked lowest systolic blood pressure and maximum heart rate until index angiography, respectively. Time-to-angiography defined the delay (in hours and minutes) between hospital admission (arrival at the emergency department) of outpatients or presentation with acute GI bleeding symptoms (melena, hematochezia, and hematemesis) of inpatients and performance of the index angiography. Technical success was defined as the cessation of extravasation on the post-embolization angiogram and the ability to perform embolization only in the presence of active contrast medium extravasation. Meanwhile, clinical success was defined as the technical success of the clinical cessation of bleeding in the patient, without early re-bleeding and further surgical, endoscopic, or repeated angiographic procedures within 30 days after angiographic embolization. Rebleeding was defined as bleeding with a drop in hemoglobin level of > 2 g/L, and/or a lack of effectiveness of conservative medical treatment. Death occurring within the hospital admission or up to 30 days post index acute GI bleeding was evaluated. Blind embolization was defined as nonselective embolization in the absence of active contrast medium extravasation and was guided by the findings of endoscopy and/or computed tomography (CT) angiography regarding the location of the bleeding vessel or by clips placed around the bleeding site [10]. Blind embolization of the vessels supplying the area of concern on endoscopy and/or CT angiography was performed if no contrast medium extravasation was observed. Additionally, in cases of endoscopically guided clip placement, the branches terminating at each clip were selectively catheterized and embolized (Fig. 1).

### Statistical analyses

The chi-square test or Fisher’s exact test was used to test for associations among various categorical variables, and the independent-samples t-test was used for non-categorical variables. Multiple logistic regression analysis was used to assess the factors associated with angiographic visualization of extravasation. All potential predictive factors with a probability value ≤ 0.05 on univariate analyses were entered into the multiple logistic regression analysis. Odds ratios (ORs), together with 95% confidence intervals (CIs), were calculated to estimate the relative risk of angiographic visualization of extravasation. Statistical analyses were performed using SPSS software (version 26.0; SPSS, Chicago, IL), and *p* < 0.05 was considered statistically significant.

## Results

### Baseline characteristics according to the angiographic visualization of extravasation

Of the 138 patients, 58 (42.0%) were with angiographic visualization of extravasation with mean time-to-angiography of 7.3 h. Table 1 summarizes the clinical and procedural features of patients with acute GI bleeding according to the angiographic visualization of extravasation. The angiographic visualization of extravasation was significantly higher in patients with diabetes (*p* = 0.036), a low platelet count (*p* = 0.048), high maximum heart rate (*p* = 0.002) and AIMS65 score (*p* = 0.026), and short time-to-angiography (*p* = 0.031). An upper GI bleeding was more likely to demonstrate angiographic visualization of extravasation than a lower GI bleeding (*p* = 0.025). Antithrombotic agent (antiplatelet agent and anticoagulants) use and initial vital signs were not associated with the angiographic visualization of extravasation. Other characteristics showed no significant difference between both groups.

**Table 1:** Comparison of groups according to the angiographic visualization of extravasation

Characteristics	Positive angiogram (n = 58)	Negative angiogram (n = 80)	*P* value
Age, mean (SD), years	66.2 (15.4)	66.8 (14.3)	0.597
Sex, men, n (%)	38 (65.5)	53 (66.3)	0.929
Underlying disease, n (%)			
Hypertension	36 (62.1)	40 (50)	0.156
Diabetes	20 (34.5)	15 (18.8)	0.036
Liver cirrhosis	2 (3.4)	4 (5)	0.985
Chronic renal disease	8 (13.8)	6 (7.5)	0.356
Chronic lung disease	3 (5.2)	5 (6.25)	0.999
Coronary artery disease	10 (17.2)	14 (17.5)	0.968
Cerebrovascular disease	12 (20.7)	12 (15)	0.384
Malignancy	12 (20.7)	27 (33.8)	0.093
Antiplatelet agent, n (%)	23 (39.7)	26 (32.5)	0.386
Anticoagulant, n (%)	9 (15.5)	9 (11.3)	0.463
Initial laboratory parameters			
Hb, mean (SD), g/dL	8.5 (2.1)	8.6 (2.7)	0.761
PLT count, mean (SD), × 10^3^/µL	193.5 (115.7)	233.4 (113)	0.048
PT, mean (SD), INR	1.7 (1.1)	1.4 (1.0)	0.150
Initial vital signs			
Systolic BP, mean (SD), mmHg	109 (24.9)	110 (20.4)	0.961
HR, mean (SD), beats per minute	96.2 (19.6)	92.9 (18.9)	0.318
Lowest systolic BP, mean (SD), mmHg	85.0 (21.4)	89.5 (16.0)	0.182
Maximum HR, mean (SD), beats per minute	118.7 (24.8)	106.7 (20.0)	0.002
Clinical Rockcall score, mean (SD)	4.6 ± 1.6	4.2 ± 1.6	0.165
Glasgow-Blatchford score, mean (SD)	10.4 ± 3.8	9.2 ± 4.3	0.098
AIMS65 score, mean (SD)	2.2 ± 1.4	1.7 ± 0.9	0.026
Location of GI bleeding, n (%)			0.025
Upper GI bleeding	29 (50)	29 (36.3)	
Lower GI bleeding	29 (50)	43 (53.7)	
Unknown	0 (0)	8 (10)	
Time-to-angiography, mean (SD), hours	5.2 (5.3)	8.9 (13.5)	0.031

*SD* standard deviation, *Hb* hemoglobin, *PLT* platelet, *PT* prothrombin time, *INR* international normalized ratio, *BP* blood pressure, *HR* heart rate, *GI* gastrointestinal,

### Clinical outcomes according to the angiographic visualization of extravasation

**Fig. 2:**
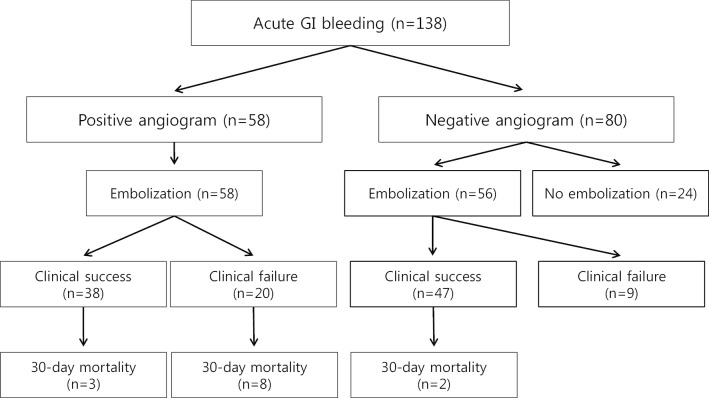
Clinical outcomes according to the angiographic visualization of extravasation

Table 2 and Fig. 2 show the clinical courses and outcomes according to the angiographic visualization of extravasation. Overall, angiographic embolization was performed in 114 of 138 (82.6%) patients who underwent initial angiographies. Of the 58 patients with angiographic visualization of extravasation, embolization was technically successful in 58 (100%) patients. All the patients showed no signs of bleeding on post-embolization angiography. Of the 80 patients without angiographic visualization of extravasation, 56 (70%) underwent blind embolization. Overall, clinical success was achieved in 85 (74.6%) patients after angiographic embolization. The clinical success rate (83.9%) of patients without angiographic visualization of extravasation was significantly higher than that (65.5%) of patients with angiographic visualization of extravasation (*p* = 0.004). The overall 30-day all-cause mortality rate of patients underwent angiographic embolization was 11.4%. The 30-day all-cause mortality rate of patients without angiographic visualization of extravasation (3.6%) was significantly lower than that (19.0%) of patients with angiographic visualization of extravasation (*p* = 0.012).

**Table 2:** Clinical outcomes according to the angiographic visualization of extravasation

Characteristics	Positive angiogram (n = 58)	Negative angiogram (n = 80)	*P* value
Successful embolization, n (%)	58 (100)	56 (70)	N/A
Clinical success, n (%)	38 (65.5)	47/56* (83.9)	0.004
30-day all-cause mortality, n (%)	11 (19.0)	2/56* (3.6)	0.012
Complication, n (%)	3 (5.2)	1/56* (1.8)	0.309

*N/A* not applicable

*Clinical success, 30-day all-cause mortality, and complication were assessed in patients underwent embolization

Altogether, there were 8 complications: 4 minor complications not resulting in significant morbidity or mortality and 4 major complications (3 bowel ischemia and 1 coil migration) resulting in significant morbidity or mortality. The three patients with bowel ischemia underwent surgical resection, while 1 patient died from massive bleeding after migration of the endovascular coils into the small bowel (coil migration). There was no significant difference between the two groups (*p* = 0.309).

### Clinical and procedural factors associated with the angiographic visualization of extravasation

Table 3 shows results of the logistic regression analysis for the clinical and procedural factors associated with the angiographic visualization of extravasation. Using multivariate analysis, time-to-angiography was the only significant factor that associated with the angiographic visualization of extravasation (*p* = 0.029). Moreover, longer time-to-angiography (hour) decreased the detection rate of an angiographic visualization of extravasation by 63% (ORs 0.373, 95% CI 0.154–0.903).

**Table 3:** Logistic regression analysis for clinical and procedural factors associated with the angiographic visualization of extravasation

Characteristics	Multivariate analysis		
	Odds ratio	95% C.I	*P* value
Diabetes	1.806	0.767–4.254	0.176
PLT count, × 10^3^/µL	0.997	0.994–1.000	0.058
Maximum HR, beats per minute	1.016	0.999–1.035	0.070
AIMS65 score	1.169	0.830–1.647	0.371
Time to angiography, hour	0.373	0.154–0.903	0.029

*C.I.* confidence interval, *PLT* platelet, *HR* heart rate

### The cutoff level of time-to-angiography that predicted the angiographic visualization of extravasation

**Fig. 3:**
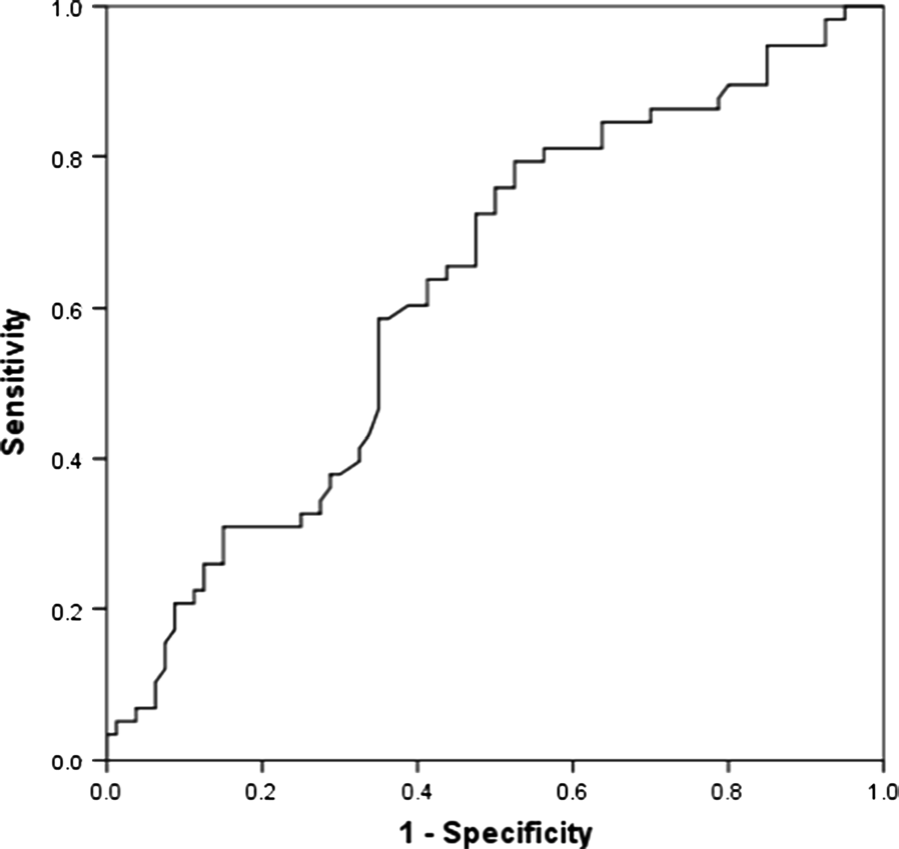
ROC curve determining the cutoff value of time-to-angiography that predicts angiographic visualization of extravasation

The receiver operating characteristic (ROC) curve determining the cutoff value of the time-to-angiography that predicted the angiographic visualization of extravasation is shown in Fig. 3. The cutoff value of the time-to-angiography was 5.0 h, with a sensitivity and specificity of 79.3 and 47.5%, respectively (*p* = 0.012). The area under the ROC curve (AUC) was 0.626.

## Discussion

Despite recent advances in endoscopic and pharmacologic therapy, acute GI bleeding remains a common and potentially life-threatening medical emergency that requires prompt diagnosis and treatment [1–3]. When endoscopic and medical management fails, angiographic embolization is not only a good alternative to surgery but also now considered the treatment of choice. However, compared to endoscopic therapy, data on the angiographic embolization in patients with acute GI bleeding, especially in optimum timeframe of angiography, are limited due to its rarity. In this study, we identified clinical and procedural predictors associated with the angiographic visualization of extravasation and clinical outcomes according to the angiographic visualization of extravasation. The time-to-angiography is the only significant factor that is associated with the angiographic visualization of extravasation, and shorter time-to-angiography increased the detection rate of angiographic visualization of extravasation. Further, patients with acute GI bleeding after angiographic embolization have different clinical outcomes and courses according to the angiographic visualization of extravasation.

Angiography plays an important role in both the diagnosis and treatment of acute GI bleeding, allowing precise localization of the bleeding site; moreover, the use of embolization or vasopressin infusion can effectively control acute GI bleeding [13, 15]. However, due to the intermittent nature of GI bleeding, the detection rate of bleeding sites using angiography has been reported to vary between 24 and 78% [13, 14]. In this study, detection rate of bleeding sites was noted in 58 (42.0%) patients with mean time-to-angiography of 7.3 h. This detection rate is slightly lower than that of previous studies, which can be attributed to the study design, excluding the indirect signs of angiography.

Active contrast medium extravasation is detectable only if the rate of arterial bleeding exceeds 0.5 ml/min [16, 17]. Therefore, the more hemodynamically unstable the patient, the greater the chance of showing the angiographic visualization of extravasation. Additionally, Lee et al. reported that blood transfusion and a drop in hemoglobin are predictors associated with the angiographic visualization of extravasation in acute GI bleeding [18]. However, to date, only a few studies have demonstrated the association between unstable vital signs and angiographic visualization of extravasation [19]. Further, the initial hemoglobin levels and blood transfusion in patients with acute GI bleeding are poor indicators for the ongoing GI bleeding. As shown in our analysis, the time-to-angiography is the only significant factor that is associated with the angiographic visualization of extravasation. In patients with short time-to-angiography, angiographic visualization of extravasation should be obtained with a high probability even when vital signs are stable or a high level of hemoglobin is maintained. Although there were studies that showed longer time-to-angiography to be a predictor of early re-bleeding after angiographic embolization, there has been no study that demonstrates the association between time-to-angiography and angiographic visualization of extravasation [11, 20].

Effective treatment requires adequate timing, although timing selection is a clinical challenge. In non-variceal upper GI bleeding, a systematic review has suggested that endoscopy within 24 h is as effective as within shorter time frames (2 or 12 h) in improving outcomes [21]. However, recent observational studies have suggested that an earlier endoscopy can be beneficial in critically ill patients, swaying most guidelines to recommend earlier endoscopy for high-risk patients, within 12 h. [22] Most patients with acute GI bleeding who require angiographic embolization are highly likely to be the high-risk patients that need earlier intervention. We analyzed the cutoff level of time-to-angiography that predicted the angiographic visualization of extravasation, and the cutoff value of the time-to-angiography was 5.0 h. However, the cutoff value of time-to-angiography is the need for continuous availability of an experienced interventional radiologist and endoscopist, which is easy to achieve only in high-volume medical centers. Moreover, the cutoff value of time-to-angiography had insufficient sensitivity and specificity that can apply to clinical settings. Further studies to examine the optimum timing and resource utilization for angiography are therefore needed.

Nearly all recent series have reported a technical success rate of angiographic embolization of greater than 90% [11, 23, 24]. However, the rate of clinical success was diverse because definition of clinical success and patient selection showed differences according to the study, and clinical success rates of 51–94% [23, 25, 26]. In this study, angiographic embolization was successful in all 58 angiograms, with angiographic visualization of extravasation (100%). Moreover, the clinical success was achieved in 74.6% (85/114) patients who underwent angiographic embolization. However, in contrast to our expectation, the clinical success was shown as a significant difference according to the angiographic visualization of extravasation. The clinical success rate of patients with active extravasation (65.5%) was significantly lower than that of patients without active extravasation (83.9%). Kim et al. reported that most patients (80%, 60/75) with negative angiographic findings such as non-extravasation have experienced spontaneous resolution of their condition without re-bleeding [27]. Although many patients without angiographic visualization of extravasation underwent blind embolization (70%, 56/80), the difference in clinical success rate between both groups reflects the natural course of the condition.

Patients with angiographic visualization of extravasation show a marked decrease in mortality when angiographic embolization is successful, compared with patients requiring surgery after failed embolization (38 vs. 83%) [28]. In this study, the overall 30-day all-cause mortality rate of patients with angiographic embolization was 11.4%. Although our result showed lower mortality rate compared to previous studies, the mortality rate is strongly correlated with clinical success according to angiographic visualization of extravasation. The 30-day all-cause mortality rate (19.0%) of patients with angiographic visualization of extravasation was significantly higher than that (3.6%) of patients without angiographic visualization of extravasation. Like clinical success, mortality rate might reflect the natural course of patients` condition. Patients with angiographic visualization of extravasation have more serious bleeding. Further, it is highly likely that the deteriorated physiologic status of patients by serious bleeding contributed to the high morbidity and mortality in patients with angiographic visualization of extravasation.

Our study has several limitations. First, the analysis had a retrospective and nonrandomized design, which is associated with the possibility of exclusion and selection bias; as may be expected in patients with more serious bleeding, there is an inevitable selection bias. Second, the sample size of our study was relatively small, which in turn limited the power of our analyses. Third, although there are important differences regarding the decision-making process for acute upper and lower GI bleeding, we included both upper and lower GI bleeding in the analysis. Patients with upper GI bleeding generally undergo urgent endoscopy for diagnosis and hemostasis before angiography; however, in patients with lower GI bleeding, urgent colonoscopy is limited to use as an initial modality in hemodynamically unstable patients (allowing for adequate bowel preparation). Despite these limitations, this study remains important for examining predictors of angiographic visualization of extravasation and characterizing the outcomes of angiographic embolization according to the angiographic visualization of extravasation.

## Conclusion

In conclusion, based on our findings, angiography timing is an important factor that is associated with the angiographic visualization of extravasation in patients with acute GI bleeding. Moreover, shorter time-to-angiography increases the detection rate of angiographic visualization of extravasation. Clinical success and mortality of patients with acute GI bleeding after angiographic embolization have different clinical outcomes and courses according to the angiographic visualization of extravasation.

## Data Availability

The datasets generated and analyzed in this article are not publicly available due to health privacy concerns. However, they are available from the corresponding author and will be obtainable by the public when the database construction is complete.

## Abbreviations

AUC: Area under the ROC curve
CIs: Confidence intervals
CT: Computed tomography
GI: Gastrointestinal
IMA: Inferior mesenteric artery
ORs: Odds ratios
ROC: Receiver operating characteristic
SMA: Superior mesenteric artery

## References

[1] van Leerdam ME, Vreeburg EM, Rauws EA, Geraedts AA, Tijssen JG, Reitsma JB, Tytgat GN (2003). Acute upper GI bleeding: did anything change? Time trend analysis of incidence and outcome of acute upper GI bleeding between 1993/1994 and 2000. Am J Gastroenterol.

[2] Prakash C, Zuckerman GR (2003). Acute small bowel bleeding: a distinct entity with significantly different economic implications compared with GI bleeding from other locations. Gastrointest Endosc.

[3] Sanders DS, Perry MJ, Jones SG, McFarlane E, Johnson AG, Gleeson DC, Lobo AJ (2004). Effectiveness of an upper-gastrointestinal haemorrhage unit: a prospective analysis of 900 consecutive cases using the Rockall score as a method of risk standardisation. Eur J Gastroenterol Hepatol.

[4] Sacks HS, Chalmers TC, Blum AL, Berrier J, Pagano D (1990). Endoscopic hemostasis. An effective therapy for bleeding peptic ulcers. JAMA.

[5] Jensen DM (1998). Diagnosis and treatment of patients with severe hematochezia: a time for change. Endoscopy.

[6] Jensen DM, Machicado GA, Jutabha R, Kovacs TO (2000). Urgent colonoscopy for the diagnosis and treatment of severe diverticular hemorrhage. N Engl J Med.

[7] Cooper GS, Chak A, Way LE, Hammar PJ, Harper DL, Rosenthal GE (1999). Early endoscopy in upper gastrointestinal hemorrhage: associations with recurrent bleeding, surgery, and length of hospital stay. Gastrointest Endosc.

[8] Rockall TA, Logan RF, Devlin HB, Northfield TC (1995). Incidence of and mortality from acute upper gastrointestinal haemorrhage in the United Kingdom. Steering Committee and members of the National Audit of Acute Upper Gastrointestinal Haemorrhage. BMJ.

[9] Kramer SC, Gorich J, Rilinger N, Siech M, Aschoff AJ, Vogel J, Brambs HJ (2000). Embolization for gastrointestinal hemorrhages. Eur Radiol.

[10] Loffroy R, Rao P, Ota S, De Lin M, Kwak BK, Geschwind JF (2010). Embolization of acute nonvariceal upper gastrointestinal hemorrhage resistant to endoscopic treatment: results and predictors of recurrent bleeding. Cardiovasc Intervent Radiol.

[11] Loffroy R, Guiu B, D'Athis P, Mezzetta L, Gagnaire A, Jouve JL, Ortega-Deballon P, Cheynel N, Cercueil JP, Krause D (2009). Arterial embolotherapy for endoscopically unmanageable acute gastroduodenal hemorrhage: predictors of early rebleeding. Clin Gastroenterol Hepatol.

[12] Loffroy R, Falvo N, Nakai M, Pescatori L, Midulla M, Chevallier O (2019). When all else fails - Radiological management of severe gastrointestinal bleeding. Best Pract Res Clin Gastroenterol.

[13] Hastings GS (2000). Angiographic localization and transcatheter treatment of gastrointestinal bleeding. Radiographics.

[14] Burke SJ, Golzarian J, Weldon D, Sun S (2007). Nonvariceal upper gastrointestinal bleeding. Eur Radiol.

[15] Jae HJ, Chung JW, Jung AY, Lee W, Park JH (2007). Transcatheter arterial embolization of nonvariceal upper gastrointestinal bleeding with N-butyl cyanoacrylate. Korean J Radiol.

[16] Sos TA, Lee JG, Wixson D, Sniderman KW (1978). Intermittent bleeding from minute to minute in acute massive gastrointestinal hemorrhage: arteriographic demonstration. AJR Am J Roentgenol.

[17] Van Beers B, Roche A (1989). Arteriography in digestive hemorrhage. Acta Gastroenterol Belg.

[18] Lee L, Iqbal S, Najmeh S, Fata P, Razek T, Khwaja K (2012). Mesenteric angiography for acute gastrointestinal bleed: predictors of active extravasation and outcomes. Can J Surg.

[19] Nakasone Y, Ikeda O, Yamashita Y, Kudoh K, Shigematsu Y, Harada K (2007). Shock index correlates with extravasation on angiographs of gastrointestinal hemorrhage: a logistics regression analysis. Cardiovasc Intervent Radiol.

[20] Walsh RM, Anain P, Geisinger M, Vogt D, Mayes J, Grundfest-Broniatowski S, Henderson JM (1999). Role of angiography and embolization for massive gastroduodenal hemorrhage. J Gastrointest Surg.

[21] Tsoi KK, Ma TK, Sung JJ (2009). Endoscopy for upper gastrointestinal bleeding: how urgent is it?. Nat Rev Gastroenterol Hepatol.

[22] Lim LG, Ho KY, Chan YH, Teoh PL, Khor CJ, Lim LL, Rajnakova A, Ong TZ, Yeoh KG (2011). Urgent endoscopy is associated with lower mortality in high-risk but not low-risk nonvariceal upper gastrointestinal bleeding. Endoscopy.

[23] Poultsides GA, Kim CJ, Orlando R, Peros G, Hallisey MJ, Vignati PV (2008). Angiographic embolization for gastroduodenal hemorrhage: safety, efficacy, and predictors of outcome. Arch Surg.

[24] Schenker MP, Duszak R, Soulen MC, Smith KP, Baum RA, Cope C, Freiman DB, Roberts DA, Shlansky-Goldberg RD (2001). Upper gastrointestinal hemorrhage and transcatheter embolotherapy: clinical and technical factors impacting success and survival. J Vasc Interv Radiol.

[25] Holme JB, Nielsen DT, Funch-Jensen P, Mortensen FV (2006). Transcatheter arterial embolization in patients with bleeding duodenal ulcer: an alternative to surgery. Acta Radiol.

[26] Loffroy R, Guiu B, Cercueil JP, Lepage C, Latournerie M, Hillon P, Rat P, Ricolfi F, Krause D (2008). Refractory bleeding from gastroduodenal ulcers: arterial embolization in high-operative-risk patients. J Clin Gastroenterol.

[27] Kim JH, Shin JH, Yoon HK, Chae EY, Myung SJ, Ko GY, Gwon DI, Sung KB (2009). Angiographically negative acute arterial upper and lower gastrointestinal bleeding: incidence, predictive factors, and clinical outcomes. Korean J Radiol.

[28] Dempsey DT, Burke DR, Reilly RS, McLean GK, Rosato EF (1990). Angiography in poor-risk patients with massive nonvariceal upper gastrointestinal bleeding. Am J Surg.

